# Accessibility Recommendations of Interfaces Designed for Individuals with Mental and Physical Disabilities: A Systematic Review

**DOI:** 10.3390/healthcare14131968

**Published:** 2026-07-02

**Authors:** Haneen Ali, Bahar Zarei, Charles Kullen, Duha Ali

**Affiliations:** 1 Mechanical and Industrial Engineering Department, Applied Science Private University, Amman 11937, Jordan; haneenbasheer@icloud.com; 2 Industrial and Manufacturing Engineering Department, California Polytechnic State University, San Luis Obispo, CA 93407, USA; zarei.bhr@gmail.com (B.Z.); ckullen@calpoly.edu (C.K.)

**Keywords:** accessibility, digital interfaces, disabilities, healthcare, inclusive design

## Abstract

**Highlights:**

This study presents a systematic review of digital healthcare interface accessibility for individuals with mental and physical disabilities, analyzing 32 selected articles from an initial pool of 2917. The findings reveal that many healthcare platforms remain insufficiently accessible due to poor compliance with accessibility standards, limited support for assistive technologies, and weak adoption of user-centered design practices. The review identifies five key themes—accessibility features, user experience, design principles, technical recommendations, and user involvement—highlighting the importance of intuitive design, personalization, and continuous user feedback. Overall, the paper emphasizes that improving accessibility requires a comprehensive, user-centered approach to reduce the digital divide and enhance equitable access to healthcare services.

**What are the main findings?**

- Many healthcare interfaces lack proper compliance with accessibility standards (e.g., WCAG), limiting usability for individuals with disabilities.
- There is a clear digital divide, with individuals with disabilities using eHealth services less and facing more challenges.
- Effective accessibility depends on user-centered design, including intuitive navigation, personalization, and integration of assistive technologies.

**What are the implications of the main findings?**

- Developers must treat accessibility as a core design requirement, integrating adaptive and inclusive features from the start.
- Policymakers should enforce stronger regulations and standardization to ensure equitable access across healthcare platforms.
- Future research should focus on evaluating real-world usability and advancing AI-driven accessibility solutions.

**Abstract:**

**Background**: The global prevalence of disabilities highlights the urgent need for accessible digital interfaces, particularly in healthcare. **Methods**: A systematic literature review was conducted to examine the current state of digital accessibility in healthcare interfaces, analyzing 32 scholarly articles to identify key challenges and recommendations for improvement. The selection process was rigorous, ensuring a comprehensive overview of existing research on accessibility in digital healthcare platforms. **Results**: Our research highlights significant issues such as the digital divide faced by individuals with disabilities and the need for inclusive design to enhance usability and accessibility. Key challenges identified include inadequate compliance with accessibility standards, limited user-centered design practices, and insufficient integration of assistive technologies. This study synthesizes best practices for creating accessible healthcare applications and websites, focusing on addressing these challenges. **Conclusions:** The authors highlight strategic recommendations aimed at ensuring that digital healthcare systems are inclusive and effective, enhancing accessibility and quality of life for individuals with disabilities.

## 1. Introduction

According to the Centers for Disease Control and Prevention (CDC) and the World Health Organization (WHO), people with disabilities represent a significant portion of the global population. The CDC approximates that 28.7% of adults in the United States, equating to roughly one in four, live with various disabilities, including impairments related to mobility, cognition, hearing, vision, and self-care [1]. Globally, the World Health Organization (WHO) estimates that over 1 billion people, or about 16% of the world’s population, experience some form of disability [2]. This necessitates accessible digital interfaces to ensure equitable access to healthcare services. Furthermore, about 25% of adults between 18 and 44 years old living with disabilities lack a consistent healthcare provider, while around 20% encounter unmet healthcare needs due to financial limitations within the previous year [1]. These statistics highlight the critical need for accessible digital interfaces to ensure equitable access to essential services, including healthcare.

Research consistently highlights the existence of a digital divide affecting individuals with disabilities and their access to digital healthcare services. Dobransky and Hargittai, 2006, highlighted that “people with disabilities are less likely to live in households with computers, are less likely to use computers, and are less likely to be online” [3]. Similarly, a survey conducted by Pettersson et al., 2023, investigated the accessibility and usage of eHealth services among individuals with and without impairments and found that individuals with impairments used eHealth services less frequently and encountered more challenges than those without impairments [4]. A multiple case study published by Fortune et al., 2024 on the use of digital technologies for providing disability services remotely during the COVID-19 pandemic also identified several barriers to effective implementation, including the complexity of accessing online platforms and poor design quality [5]. Together, these studies highlight persistent accessibility barriers and the need for more inclusive digital healthcare systems [3,4,5].

Established guidelines for accessible interfaces can significantly enhance the quality of life for people with disabilities, especially in healthcare [6,7]. A systematic review by Ara et al., 2023 [8], emphasizes the importance of following established guidelines, such as the Web Content Accessibility Guidelines (WCAG), to ensure digital accessibility. These guidelines provide a comprehensive framework for developing user-friendly and accessible digital interfaces [8,9,10]. “Inclusive Design for a Digital World: Designing with Accessibility in Mind,” Regine Gilbert, 2019, [10] emphasizes adhering to the WCAG to ensure digital accessibility. WCAG 2.1 includes new criteria to address mobile accessibility, support for users with low vision, and cognitive and learning disabilities. These guidelines aim to make digital content more perceivable, operable, understandable, and robust, ensuring that all users’ needs and preferences are considered. Gilbert discusses common accessibility challenges, such as using animated text and Emoji icons, which can be problematic for users relying on assistive technologies like speech synthesizers [10].

In summary, designing inclusive and accessible digital interfaces is crucial for ensuring that people with disabilities can effectively use healthcare portals and other essential digital platforms. By incorporating established accessibility guidelines and involving users with disabilities in the design process, developers can create more inclusive and user-friendly interfaces. This systematic literature review aims to explore recent accessibility recommendations for interfaces designed for individuals with mental or physical disabilities, with a particular emphasis on healthcare portals and mobile app design. The findings will provide valuable insights into best practices for developing accessible digital platforms, ultimately enhancing the quality of life for people with disabilities. By synthesizing the current literature, this study highlights these gaps and provides actionable recommendations for inclusive interface design.

## 2. Methodology

### 2.1. Research Questions

The objective of this systematic literature review is to explore the recent accessibility recommendations of interfaces designed for individuals with mental or physical disabilities, with a particular emphasis on existing healthcare portals. The research aims to answer the following questions:

1. What are the current interface design recommendations for people with disabilities?

This question seeks to investigate existing interfaces across various platforms and their accessibility features for the disabled community. The goal is to identify any existing recommendations and suggestions for design improvements that could enhance accessibility and quality of life for people with special needs. This includes evaluating the usability, accessibility features, and overall design principles that are recommended or currently in use.

2. What are the current healthcare portals and mobile apps design recommendations for people with disabilities?

This question focuses specifically on healthcare portals and mobile app designs, examining how accessible they are to individuals with mental or physical disabilities. The purpose is to uncover current design suggestions and address the concerns and issues faced by these individuals. This involves analyzing the user interface (UI) and user experience (UX) design elements that cater to accessibility, such as text readability, navigation simplicity, and assistive technology compatibility.

### 2.2. Search Strategy

To gather relevant literature, a comprehensive search strategy was employed. This involved the use of a set of keywords, as illustrated in Table 1. The search was conducted across three major academic databases known for their extensive collection of peer-reviewed articles: PubMed, ScienceDirect, and Web of Science. Web of Science was chosen because it covers a wide range of high-quality research in many different areas, including health technology and user experience. ScienceDirect was selected due to its extensive repository of peer-reviewed journals, particularly in the domains of computer science, healthcare technology, and human–computer interaction, ensuring access to relevant research. Lastly, PubMed, a trusted resource in the medical and health sciences community, was included to capture the latest advancements and empirical studies in healthcare interface design and accessibility, providing a critical medical perspective to the review. The combination of these three databases ensures a thorough and credible examination of the literature, encompassing a wide range of disciplines essential to understanding design recommendations for individuals with disabilities. The complete database-specific search strategies, including Boolean operators and filters, are provided in Appendix A.

### 2.3. Articles Inclusion and Exclusion Criteria

To refine the search results, specific inclusion and exclusion criteria, as shown in Table 2, were established. Peer-reviewed articles published in English within the last ten years, focusing on interface design and accessibility for individuals with disabilities, were included. Articles that did not have the full text accessible or were not directly related to this study’s topic were excluded. Non-peer-reviewed articles, studies not available in full text, publications in languages other than English, and research not directly related to human interface accessibility were excluded.

### 2.4. Screening Articles

To ensure unbiased results, a structured screening method was utilized. The articles were uploaded to Rayyan ai (https://www.rayyan.ai/), a research collaboration platform developed by the Qatar Computing Research Institute (QCRI) in Qatar. The first step involved removing duplicate entries. Each researcher then conducted an initial round of screening, where they reviewed the articles and labeled them as either included, not certain, or not included if the paper was not related to the field of study. After this first round, the researchers reviewed the “not certain” articles again to make a final decision.

**Figure 1:**
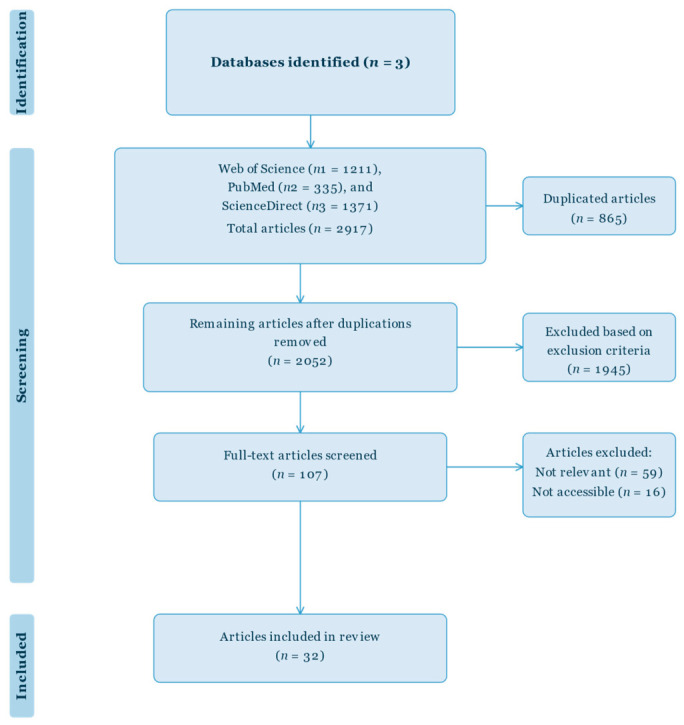
PRISMA flow diagram illustrating the study selection process for the systematic review of accessibility in digital healthcare interfaces.

Figure 1 displays the PRISMA flow diagram of the methodology used for searching. Initially, a total of 2917 articles were found through the three databases: Web of Science, PubMed, and ScienceDirect. After removing 865 duplicates, the abstracts of the remaining 2052 articles were screened. Based on the exclusion criteria, 1945 studies were removed. The full texts of the remaining 107 articles were reviewed, which were evaluated for their relevance to the topic of accessibility features in interface designs for individuals with mental and physical disabilities. This final assessment led to the exclusion of 74 articles, leaving 32 that met all the criteria. This review was conducted following PRISMA guidelines; however, a formal review protocol was not registered.

### 2.5. Data Analysis and Theme Development

This study uses a qualitative synthesis approach to analyze the selected literature. Due to the variability in study designs, methods, and reported outcomes, a quantitative meta-analysis or statistical comparison was not appropriate for this review. A formal appraisal tool such as CASP was not applied; the evaluation approach used in this review allowed for consistent comparison using relevance and methodological clarity. This approach aligns with PRISMA recommendations for transparency in reporting potential sources of bias.

Data extraction and theme identification were conducted by one researcher across all 32 included studies. The extracted data and emerging themes were subsequently reviewed and discussed collaboratively among the research team to ensure consistency and alignment with the study objectives. Themes were iteratively refined throughout the analysis process as patterns became apparent across the included literature. Because coding was performed by a single researcher, formal inter-rater reliability measures such as Cohen’s kappa were not calculated, which represents a limitation of the synthesis.

### 2.6. Quality Assessment and Risk of Bias

To ensure the reliability of the findings, the quality of the included studies was considered throughout the review process. Although a formal scoring method was not used, each study was evaluated based on its relevance to the research questions, clarity of methodology, and overall contribution to understanding accessibility in digital healthcare interfaces. Differences in study design, sample size, and focus were observed across the selected articles, which may influence how broadly the findings can be applied. This evaluation involved reviewing each study’s design, objectives, and reported findings, allowing for consistent comparison across the included articles. While the studies varied in methodology, the use of peer-reviewed sources and clear inclusion criteria supports the overall reliability of the findings.

Potential sources of bias were also considered. The review was limited to peer-reviewed articles published in English, which may introduce language bias and exclude relevant studies in other languages. In addition, the use of specific databases (PubMed, ScienceDirect, and Web of Science) may have limited the range of studies included. Publication bias may also be present, as studies with more significant findings are more likely to be published. These factors were considered when interpreting the results of this review.

To increase transparency regarding the quality of the included studies, each article was evaluated using three criteria: relevance to the research questions, methodological clarity, and overall contribution to the review. Relevance assessed how directly the study addressed accessibility, disability, healthcare interfaces, or related design recommendations. Methodological clarity evaluated whether the study design, data collection methods, and analysis procedures were clearly described. Contribution reflected the extent to which the study provided meaningful findings, recommendations, or insights relevant to accessible digital healthcare interfaces.

Studies were assigned ratings of High, Moderate, or Low for each criterion. These ratings were intended to provide a general assessment of the evidence base and support interpretation of the findings rather than serve as a formal risk-of-bias evaluation. The legend for ratings is provided in Table 3, and a summary of these assessments is provided in Table 4.

## 3. Results and Discussion

The review of 32 articles identified five key themes to enhance the quality of systematic literature on accessibility: Accessibility Features, User Experience, Design Principles, Technical Recommendations, and User Feedback and Participation. Emphasizing compliance with accessibility standards, user-friendly designs, and support for assistive technologies, the review highlights the importance of intuitive navigation, personalization options, and feedback mechanisms. User-centered design, consistent layouts, and clear instructions are essential design principles. Technical recommendations focus on adaptive interfaces, robust error handling, and maintaining security and confidentiality. Involving users with disabilities in the design and testing phases is crucial for addressing potential issues and ensuring the interface meets their needs. The advantages and disadvantages of these themes illustrate a balanced approach to creating accessible interfaces, acknowledging both potential and challenges. All contributing articles are cited in Table 5.

Table 5 summarizes the key accessibility recommendations identified across the included studies, with an emphasis on usability, user-centered design, and technical support. While the table provides a detailed overview, the results highlight recurring patterns across these themes.

### 3.1. Accessibility Features

**Ensure Compliance with Accessibility Standards (19 occurrences):** This category encompasses articles emphasizing the importance of following established accessibility guidelines such as the Web Content Accessibility Guidelines (WCAG) to ensure interfaces are usable by people with various disabilities. Accessibility standards are critical in several contexts. For example, a study by Van Cleave et al., 2022, highlights that ensuring that telehealth interfaces that are accessible can help mitigate disparities in care for children and youth with special health care needs (CYSHCN). Studies indicate that accessible telehealth technology can address known gaps in care by making services more equitable and effective for these populations [11]. Similarly, an article by Garling and Stewart, 2024 states that enhancing inclusivity and creating stigma-free environments in health communication also depends on compliance with accessibility standards. This approach ensures that healthcare services are welcoming and usable for all patients, including those with disabilities [12].

Further supporting this notion, Hemsley et al., 2018, reported that E-health literacy is another area where accessibility is paramount. Inclusive electronic Personal Health Records (e-PHR) systems must consider personal factors and follow accessibility guidelines to serve diverse populations effectively. Ensuring these systems are accessible can significantly enhance the usability and effectiveness of digital health records [13]. In addition to improving WCAG compliance for individuals with physical disabilities, a study by Onyeaka et al., 2022, stated that in mental health, addressing language barriers and optimizing patient portals for smartphone access are essential for equitable healthcare delivery. Studies suggest that following accessibility standards in these areas can lead to better engagement and outcomes for diverse populations, including those who are smartphone-dependent or have language needs [14].

Moreover, Ramos et al., 2021, noted that in the realm of app development, incorporating Diversity, Equity, and Inclusion (DEI) factors is crucial. Evaluating and designing mental health apps with these factors in mind ensures they are accessible to all users, including those from marginalized communities. By adhering to accessibility standards, developers can create apps that meet the diverse needs of their users [15]. Another example illustrating the importance of accessibility in digital technology was mentioned by Senjam & Primo, 2022, who discussed the use of screen readers and text-to-speech functions in smartphones. These features enable individuals with visual impairments to use smartphones effectively, highlighting the broader application of accessibility standards in everyday technology [16]. These examples collectively illustrate the widespread recognition of the importance of accessibility standards in ensuring equitable access and usability for all individuals, including those with disabilities. By adhering to established guidelines, digital health tools and services can become more inclusive, supporting the diverse needs of all users.

Overall, these findings show that accessibility standards are consistently recognized as an essential part of designing digital healthcare platforms. This recurring theme suggests that following guidelines such as WCAG remains a key priority across different contexts. This theme was reported across 19 of 32 included studies, making it a widely represented finding. However, multiple studies also point out that these standards are not always fully implemented in practice, highlighting an ongoing gap between real-world application and what is recommended in guidelines.

**User-Friendly Designs (3 occurrences):** This category emphasizes the importance of designing interfaces that are simple, clear, and easy to navigate, with features like large buttons, high-contrast text, and alternative text for images. Various articles highlight the need for user-friendly designs in different contexts. Buning et al., 2024, reported that, for instance, in healthcare settings, it is crucial to accommodate patients’ specific needs by providing options such as large print, assistance with transfers, and help with filling out forms. This approach ensures that interfaces and workflows are accessible and easy to use for all patients, regardless of their disabilities [17]. Similarly, as mentioned by Jo et al., 2021, creating accessible and informative public health websites is essential for individuals with disabilities, as seen in the context of COVID-19 vaccine information. Eliminating barriers like CAPTCHA, which stands for completely automated public Turing test, to tell computers and humans apart and providing alternative access methods such as direct phone numbers and transportation options demonstrate the need for clear and navigable designs [18]. Additionally, another study, Berkowsky and Czaja 2018, stated that technology training courses must cater to the needs of older learners and individuals with physical or cognitive limitations. Suggestions include seating participants with vision or hearing issues close to the front, using a microphone, and providing customized equipment like large trackball mice and high-resolution screens. Presenting content in multiple formats, including step-by-step manuals with screenshots, further supports the creation of user-friendly designs that are accessible to all users [19]. These examples, displayed across three articles, highlight the importance of designing interfaces that are not only functional but also inclusive and easy to use for people with various disabilities.

**Support for Assistive Technologies (5 occurrences):** This category highlights the necessity for interfaces to be compatible with screen readers, voice recognition software, and other assistive technologies to facilitate access for users with disabilities. For instance, Van Holstein et al., 2021, found that in library services, the need for staff to assist users, especially those with intellectual disabilities, underscores the importance of supporting assistive technologies to bridge digital access gaps [20]. Similarly, Cosgrove et al., 2023, reported that designing mHealth applications compatible with both iOS and Android platforms ensures broader accessibility, allowing users with different devices to benefit from the applications’ features [21]. Moreover, Zhou et al., 2020, highlighted the significance of customizable accessibility features in the iMHere 2.0 app, which improved usability for participants with dexterity issues. This is another example of the importance of supporting assistive technologies tailored to users’ specific needs in mHealth apps [22]. With five instances, these articles suggest moderate support for the role of assistive technologies in making digital platforms and applications accessible to individuals with various disabilities.

### 3.2. User Experience

**Intuitive Navigation (24 occurrences):** This category emphasizes the importance of designing interfaces with intuitive navigation, ensuring users can easily find the information they need without unnecessary complexity. Various studies highlight the role of intuitive navigation in different contexts. For instance, a study conducted in Saudi Arabia by Jabour et al., 2022, explains the need for an efficient and smooth appointment-making system, which underscores the importance of intuitive navigation in healthcare settings. Users prefer systems that allow them to easily book appointments and receive necessary information, reflecting a demand for clear and straightforward navigation [23].

In the realm of telehealth, Van Cleave et al., 2022, reported that for children and youth with special health care needs (CYSHCN), incorporating these technologies effectively implies designing interfaces that are easy to navigate. This ensures that users, particularly those with difficulties accessing in-person care, can easily utilize telehealth services [11]. Similarly, as mentioned by Van Holstein et al., 2021, libraries and digital services benefit from maintaining staffed check-out counters and having intermediaries assist users, highlighting the importance of intuitive navigation. This approach ensures that individuals can easily access the services they need despite potential digital barriers [20].

Optimizing patient portals for smartphone access is crucial, particularly for addressing language barriers and improving engagement among diverse populations. Onyeaka et al., reported that this further emphasizes the need for intuitive navigation, ensuring that users can easily access and interact with these portals [14]. As mentioned by Cosgrove et al., 2023, designing m-health applications with features like storing provider visit summaries and tracking health data indicates the importance of intuitive navigation, allowing users to manage their health information effortlessly [21].

Arean et al., 2016, noted that ensuring mobile apps for moderate depression are easy to navigate is vital for user engagement and effectiveness, particularly for features targeting cognitive correlates of depression [28]. As Salgado et al., 2018, reported, the consensus on important features for a medication management app, such as automatic refills and sharing medication information, implies the need for intuitive navigation to help users easily access and use these functionalities [30]. Lastly, the potential of eHealth tools to support patients with chronic complex diseases and disabilities in community and primary care settings indicates the need for intuitive navigation. Easy-to-use interfaces support ongoing patient-provider interaction and effective self-management, as mentioned by Steele Gray et al., 2014 [31]. These examples demonstrate the critical role of intuitive navigation in various applications, ensuring that users can easily access and utilize digital tools and services effectively.

These findings suggest that intuitive navigation is a key aspect of accessible interface design in digital healthcare. Appearing across 24 of the 32 included studies, it represents one of the most frequently reported themes, indicating that navigation-related usability challenges remain a widespread barrier in digital healthcare systems. For example, in scheduling appointments or accessing health information, complex navigation can limit effective use of these platforms. This is especially important for individuals with cognitive, language, or motor impairments.

**Personalization Options (10 occurrences):** This category highlights the importance of allowing users to customize their interfaces to enhance their experience. Various studies emphasize the need for personalization in different contexts. For instance, Hemsley et al., 2018, found that designing e-health literacy interventions for different populations requires customization in electronic Personal Health Records (e-PHR) systems to ensure inclusivity and user-friendliness [13]. Similarly, Onyeaka et al., 2022, noted that online health communication platforms must address cultural, ethnic, demographic, and economic diversity, offering personalization options to improve engagement and equitable access [14]. Moreover, Cosgrove et al., 2023, reported that mHealth applications should include features like age-specific health information, tailoring the interface to user needs for relevance and effectiveness [21]. In the realm of technology training, Berkowsky and Czaja, 2018, found that older learners benefit significantly from specialized equipment and customized materials, ensuring the learning experience is adapted to their needs [19].

It is important to note that not only do applications used by patients with physical disabilities need interface customizations, but patients with mental disabilities also require the same accessibility to enhance their experience. Ramos et al., 2021, emphasized that app-based mental health care should incorporate Diversity, Equity, and Inclusion (DEI) factors into design and evaluation, considering special needs and representing marginalized identities to enhance accessibility [15]. As an example, a study conducted by Maloney et al., 2020, developed smartphone-based mental health resources for Jamaican adolescents, incorporating culturally relevant language and preferences to enhance privacy, confidentiality, and effectiveness. The study highlighted that smartphone-based health services provide scalable, sustainable, and low-stigma methods for delivering low-intensity treatments. These services, which include automated messages, interactive apps, and virtual consultations, enhance access to mental health care by enabling self-guided methods with minimal professional assistance [32]. These ten examples collectively demonstrate the critical role of flexible and customizable interfaces in various applications, ensuring services are tailored to meet diverse user needs and preferences, thereby enhancing overall user experience and accessibility. For healthcare, this is particularly important, where users of all ages have varying levels of digital literacy and different types of impairments.

**Feedback Mechanisms (13 occurrences):** This category emphasizes the importance of integrating mechanisms that allow users to provide feedback on their experiences, which is crucial for identifying areas for improvement and ensuring continuous enhancement of the portal. For instance, Jabour et al., 2022, reported that in healthcare appointment scheduling, patient preferences highlight the need for feedback mechanisms to improve communication and service quality [23]. Similarly, Garling and Stewart, 2024, emphasized that enhancing patient-provider communication and understanding underscores the importance of gathering feedback to identify and address areas needing improvement [12]. Sit et al., 2021, also noted that feedback mechanisms are particularly vital in mental health interventions, where user opinions help refine interactive sections on portals [26].

Moreover, Ramos et al., 2021, highlighted the development of DEI (Diversity, Equity, and Inclusion)-focused app evaluation frameworks, stressing the necessity of incorporating feedback from marginalized communities to continuously improve app design and evaluation [15]. This is further supported by Maloney et al., 2020, whose study on smartphone-based mental health resources for Jamaican adolescents found that understanding user perceptions and barriers is crucial for improving mHealth resources [32]. Additionally, research by Shamsujjoha et al., 2024, demonstrated that in designing human-centric eHealth apps, feedback mechanisms are crucial for gathering input from both users and developers to ensure continuous improvement [25].

This recurring emphasis across 13 articles on user feedback shows that accessibility cannot be fully achieved without ongoing user input, especially from users with disabilities. Designers may not encounter and fully understand a system from a user perspective and must be open to system evolution with time. Accessibility should be viewed as an iterative process rather than a one-time update and design solution.

### 3.3. Design Principles

**User-Centered Design (30 occurrences):** Yang et al., 2024, state that systemic accommodations for people with disabilities during pandemic planning demonstrate the importance of these principles [33]. Prioritizing app learnability for a broad range of users, including those with disabilities, is a key aspect of user-centered design. A practical application of these principles is seen in designing an application compatible with both iOS and Android platforms based on user feedback, as shown in a study by Cosgrove et al., 2023 [21]. Adding customizable accessibility features based on user feedback further aligns with these principles, as noted in research conducted by Zhou et al., 2020 [22].

Campbell and Besselli, 2020, reported that enhancing communication among direct care workers and clinicians through electronic management systems aligns with user-centered design by improving user experience and efficiency [34]. Furthermore, considering end-user needs to enhance staff efficiency and patient outcomes in private healthcare is another example of user-centered design in action. Overall, these examples demonstrate the critical role of user-centered design principles in various applications, ensuring continuous improvement and enhancing overall user experience and accessibility. This theme was identified across 30 of the 32 included studies, making it one of the most widely supported findings in this review.

**Consistent Layouts (1 occurrence)**: This category emphasizes the importance of maintaining consistent use of layouts, icons, and terminology to aid user understanding and navigation of the portal. As mentioned in a study published by Cosgrove et al., 2023, on m-health applications to support caregivers of children with Down syndrome, the need to design an application compatible with both iOS and Android platforms highlights the importance of consistent layouts in m-health applications. The results of a conducted interview identified key features such as storing provider visit summaries, contact information, caregiver notes, and tracking health data like growth charts, medications, laboratory findings, and development. While the concept of consistent layouts is not explicitly mentioned, the integration of multiple features necessitates a consistent layout to ensure users can easily understand and navigate the application. By applying consistent design principles, the m-health application can improve user experience, making it easier for users to access and manage their health information across different devices and features [21]. However, this theme was identified in only one of the 32 included studies, indicating that the evidence supporting consistent layouts as an accessibility recommendation is currently limited compared to more frequently reported themes.

**Clear Instructions and Help Options (26 occurrences):** This category emphasizes the importance of providing clear, concise instructions and accessible help options to reduce user frustration and improve overall satisfaction. Various articles highlight the necessity of clear instructions and help options in different contexts. For instance, as mentioned by Jabour et al., 2022, in healthcare appointment scheduling in Saudi Arabia, initiatives such as increasing clinic appointment options, providing online booking, and ensuring out-of-hours availability show the need for clear instructions to facilitate a smooth user experience during the transition to a web-based appointment system. Similarly, patients’ preferences for clear communication, including explanations of their health and treatment and regular updates, underscore the importance of clear instructions and help options to meet their information needs [23]. Van Cleave et al., 2022, reported that in telehealth for children and youth with special healthcare needs (CYSHCN), providing clear instructions is crucial for ensuring these users, who often face complex conditions and socioeconomic barriers, can effectively utilize telehealth services [11]. Accessibility issues, such as those faced by individuals with disabilities in accessing COVID-19 vaccine information, highlight the need for alternative methods like direct phone numbers and transportation options, demonstrating the necessity of clear instructions and help options to facilitate access, as shown in a study by Jo et al., 2021 [18].

Additionally, according to an article published by Onyeaka et al., 2022, online health communication platforms also benefit from addressing language barriers with bilingual options, reinforcing the need for clear instructions to promote user engagement [14]. Lastly, Berkowsky and Czaja, 2018, reported that technology training courses tailored to older learners’ needs, with step-by-step instructions and customized equipment, align with the necessity of clear instructions and help options to ensure effective engagement with the training content [19]. Appearing across 26 studies, this theme is very widely represented. These examples underscore the critical role of clear instructions and help options in reducing user frustration and enhancing satisfaction across various contexts.

### 3.4. Technical Recommendations

**Adaptive Interfaces (23 occurrences):** This category emphasizes the importance of designing adaptive interfaces that can adjust to different devices and user preferences, ensuring accessibility across various platforms. Various articles highlight the necessity of adaptive interfaces in different contexts. For instance, Van Cleave et al., 2022, noted that recognizing access barriers and the need for digital access points like telecom shops for people with intellectual disabilities underscores the importance of adaptive interfaces that can adjust to different user needs and environments [11].

Additionally, a study conducted by Yang et al., 2024, on disability-inclusive pandemic planning, which involves systemic accommodations at different levels, suggests designing adaptive interfaces that can adjust to various contexts and user needs [33]. Berkowsky and Czaja, 2018, reported that technology training courses for older learners emphasize using different equipment and formats to cater to various needs, implying the need for adaptive interfaces that can adjust to different devices and user preferences to ensure accessibility across platforms [19]. Continuous technological innovation in the Metaverse for healthcare also suggests the need for adaptive interfaces that can evolve with new technologies and user preferences, as shown in a study by Malik et al., 2024 [35]. The development of culturally relevant mHealth resources for Jamaican adolescents, as studied by Maloney et al., 2020, implies the need for adaptive interfaces that can adjust to different user preferences and cultural contexts, ensuring accessibility across various populations [32].

Furthermore, Du and Salen Tekinbas, 2020, reported that the collaboration between child-computer interaction designers and speech-language pathologists (SLPs) to address clinical, design, and technical challenges emphasizes the need for adaptive interfaces that can adjust to different user needs and contexts, ensuring accessibility and usability across stakeholders [36]. Bakker et al., 2018, noted that the distinct functionality of different mental health apps (MHapps) for mental health disorders indicates the need for adaptive interfaces that can adjust to various user needs and preferences, ensuring accessibility and usability across different apps [27]. Identified areas for improvement in app clarity, organization, and inclusiveness suggest the need for adaptive interfaces that can adjust to different user needs and preferences, ensuring accessibility across contexts, as shown in a study by Kairy et al., 2021 [37]. These 23 examples strongly highlight the essential role of adaptive interfaces in guaranteeing accessibility and usability across diverse platforms and user contexts.

**Robust Error Handling (4 occurrences):** This category emphasizes the importance of implementing robust error handling mechanisms to help users recover from mistakes and understand what went wrong. Various articles highlight the necessity of robust error handling in different contexts. For instance, Cosgrove et al., 2023, discuss the development of m-health applications compatible with both iOS and Android platforms. The complexity of managing health data, including provider visit summaries, contact information, and health tracking data, suggests the importance of robust error handling to help users recover from mistakes and understand errors in data management [21].

Similarly, Fabius et al., 2023, report that the use of electronic management systems for direct care workers to improve communication and reduce inefficiencies in patient care hand-offs implies the need for robust error handling to avoid miscommunication and enhance overall efficiency [24]. In private healthcare, recommendations to improve efficiency and reduce unnecessary hospital visits underscore the necessity of robust error handling to prevent mistakes and ensure system reliability, thereby improving patient outcomes and reducing healthcare costs, as mentioned by Campbell and Besselli, 2020 [34].

Finally, according to a study published by Malik et al., 2024, in the context of the Metaverse in healthcare, continuous technological innovation, robust regulatory supervision, and sound governance structures emphasize the need for robust error-handling mechanisms to ensure data security and ethical use, helping users recover from mistakes and understand what went wrong in this evolving digital landscape [35]. These examples collectively underscore the critical role of robust error handling in ensuring system reliability, efficiency, and user satisfaction across various healthcare contexts. With only four occurrences, this theme is not as widely represented compared to more frequent themes presented.

**Secure and Confidential (11 occurrences):** This category emphasizes the importance of ensuring health portals are secure and maintain user confidentiality, given the sensitive nature of health information. Various articles highlight the necessity of secure and confidential systems in different contexts. In designing and implementing online health communication platforms, concerns about data security and privacy are paramount. According to research by Onyeaka et al., 2022, efforts to improve data transparency, protection, and patient privacy are crucial to encourage engagement, particularly in mental health contexts where confidentiality is vital [14].

For direct care workers, the use of electronic management systems to share important health information between clinicians, residential service agencies, and families underscores the need for secure and confidential systems. Fabius et al., 2023, reported that these systems are essential to protect sensitive health data and enhance communication efficiency [24].

Participants’ views on utilizing the internet for mental health patients stress the importance of secure systems for exchanging personal information confidentially. Maintaining anonymity and protecting clients’ privacy is crucial for building trust and effective communication in mental health interventions, as mentioned by Sit et al., 2021 [26]. Similarly, Malik et al., 2024, noted that the potential of the Metaverse in healthcare highlights the need for robust regulatory supervision to address data security and ethical use cases, ensuring user confidentiality as it evolves to redefine traditional healthcare systems [35].

Recommendations for app evaluation frameworks to incorporate Diversity, Equity, and Inclusion (DEI) factors are crucial. Ensuring secure exchanges of personal material aligns with maintaining user confidentiality, as stated by Ramos et al., 2021 [15]. Lastly, as Shamsujjoha et al., 2024, mentioned, designing human-centric eHealth apps involves managing conflicting issues and maintaining quality, which requires secure systems to handle sensitive health information and protect user data [25].

In medication management apps, even though privacy settings and password protection were considered less critical, ensuring the app’s security and maintaining user confidentiality remains essential for handling sensitive health information, as noted by Salgado et al., 2018 [30].

These 11 examples highlight the critical role of secure and confidential systems in protecting sensitive health information, ensuring data integrity, and maintaining user trust across various healthcare contexts.

### 3.5. User Feedback and Participation

**Involve Users in the Design Process (29 Occurrences):** Actively involving users, particularly those with disabilities, in the design and testing phases can help identify potential issues and ensure the portal meets their needs. Buning et al., 2024, stated that among individuals with at least one disability, there was a nearly equal split between those who requested accommodation and those who did not. There was significant variability in the types of accommodation requested, even among those with the same type of disability. This highlights the need for health care providers and staff to avoid making assumptions about accommodations and to allow patient preferences to guide which accommodations should be provided. Primary care clinics are recommended to conduct similar surveys to identify necessary accommodations [17].

Furthermore, according to an article published by Yang et al., 2024, most requested accommodations did not require significant workflow modifications or expenses. For example, large print was a commonly requested accommodation among those with low vision or blindness. This feature is available for free in certain electronic health records, including Epic Systems. Other accommodations typically centered on how care was delivered at the clinic, requiring flexibility in care protocols (e.g., assistance with transfers, filling out forms) and communication approaches (e.g., inclusion of support persons, additional communication time between patients and providers) [33].

As Fabius et al., 2023, noted, schedulers can include questions about the presence of a disability and inquire if there are specific needs or accommodations to make appointments more accessible [24]. Disability-related questions were found not to be intrusive to patients, with no objections from patients about being asked about the presence of a disability, demonstrating the feasibility of implementing these questions in a primary care setting, as mentioned by Campbell and Besselli, 2020 [34].

These 29 examples underscore the importance of user-friendly designs, personalization options, and involving users in the design process. Recommendations for primary care clinics to survey their patients to identify required accommodations involve users directly in the design and improvement process, ensuring the portal meets their needs effectively and inclusively. The identification of this theme across 29 studies makes it one of the most strongly supported findings in this review, highlighting broad agreement on the importance of user-centered design in accessible digital healthcare interfaces.

**User Testing and Iteration (21 occurrences):** This category highlights the significance of conducting user testing sessions and iterating on the design based on user feedback to refine the interface and improve usability. Various articles emphasize the necessity of user testing and iteration in different contexts. For telehealth technologies, Van Cleave et al., 2022, reported that additional studies are needed to understand the best ways to incorporate these evolving technologies into the care of children with special health care needs. This underscores the importance of user testing and iteration to refine the technology and improve its usability [11]. Similarly, implementing recommendations from people with mobility disabilities involves iterative processes and feedback loops to refine and improve accommodations based on user experiences and feedback, as mentioned by Yang et al., 2024 [33].

Fabius et al., 2023, suggested that expanding innovative technologies and investing in messaging capabilities for direct care workers requires continuous user testing and iteration to address issues and improve communication [24]. Private healthcare recommendations that focus on educational campaigns and community outreach also indicate the need for continuous feedback and iteration to enhance the effectiveness of these programs, as mentioned by Campbell and Besselli, 2020 [34]. Sit et al., 2021, reported that participants’ opinions on utilizing the internet for mental health patients suggest the need for continuous user testing and iteration to refine methods such as search engines, chat tools, and videos to enhance their effectiveness [26].

According to a study by Shamsujjoha et al., 2024, plans to conduct observational studies and gather feedback on key deficiencies in eHealth apps imply the need for continuous user testing and iteration to refine these apps and enhance their effectiveness [25]. Findings that certain mobile apps have a stronger effect on depressed mood imply the need for continuous user testing and iteration to refine these apps and support mental health for people with moderate depression, as mentioned by Arean et al., 2016 [28].

Senjam and Primo, 2022, noted that encouraging the use of smartphones by people with visual impairments and addressing challenges like unreadable content and slow screen reader responsiveness requires continuous user testing and iteration to refine these devices [16]. The focus on digital health equity and continuous improvement in fostering partnerships between health consumers and providers also implies the need for ongoing user testing and iteration to refine digital health tools and support vulnerable populations, as stated by Ha et al., 2023 [38].

Considering patient-identified needs and concerns in adopting eHealth technologies suggests the need for continuous user testing and iteration to refine these tools and support patients with chronic conditions and disabilities [31]. The use of focus group feedback in developing the IIAM app for individuals with neurodevelopmental disabilities highlights the importance of continuous user testing and iteration to refine the app and enhance its effectiveness in improving the quality of life for these individuals [39]. These 21 examples underscore the critical role of conducting user testing sessions and iterating on the design based on user feedback to refine interfaces and improve usability across various applications and technologies.

The five identified themes are interconnected and together contribute to the accessibility of digital healthcare interfaces. Design principles provide the foundation for development, while user experience and accessibility features directly influence usability. Technical recommendations support the implementation of these elements, and user feedback and participation enable continuous improvement over time. This framework highlights accessibility as an ongoing and iterative process.

## 4. Implications

The findings presented in Section 3 (Results and Discussion) highlight several key factors influencing accessibility in digital healthcare interfaces. Building on these results, this section discusses their implications for design, development, and future research.

These findings reveal far-reaching implications for policymakers, interface developers, and researchers. Adhering to inclusive design principles and integrating user feedback can enhance the accessibility of healthcare systems, leading to improved quality of life for individuals with disabilities. Furthermore, these insights inform the development of policies to ensure equitable access to digital healthcare services.

## 5. Emerging Trends and Recommendations for Future Interface Design

Based on the comprehensive analysis conducted in this study, several strategic recommendations are proposed to enhance the accessibility of digital healthcare interfaces for individuals with disabilities. First, it is recommended that healthcare institutions and developers strictly adhere to established accessibility standards, such as the Web Content Accessibility Guidelines (WCAG).

Incorporating user-centered design practices is also essential. Developers should involve individuals with disabilities in design and testing to identify barriers early. Recent literature by Charalambous, 2026 also emphasizes the importance of participatory and community-centered co-design approaches, where individuals with disabilities are involved throughout the entire development and implementation process rather than only during usability testing [43]. Platforms should also support assistive technologies such as screen readers and voice recognition. Personalization and feedback mechanisms should be integrated to support diverse user needs and continuous improvement. Robust error handling mechanisms and feedback systems are also recommended to guide users through potential issues.

Moreover, the security and confidentiality of sensitive health information must be prioritized. Recent research by Jain et al., 2025, also emphasizes that digital health accessibility should be treated as a health equity and civil rights issue, particularly as healthcare systems become increasingly dependent on digital technologies and AI-driven platforms [44]. Healthcare platforms should implement strong data protection measures to maintain user trust and comply with legal and ethical standards. Future research should explore the long-term impact of these recommendations on accessibility and usability in diverse healthcare settings. Additionally, policies should be developed to ensure that these digital solutions are equitably accessible to all individuals, regardless of their technological proficiency or socioeconomic status.

It is recommended that interface designs prioritize advanced technologies like voice interfaces and AI-driven natural language processing (NLP) to enhance accessibility. These technologies enable intuitive, hands-free interactions, which are particularly beneficial for users with motor or visual impairments. For example, the integration of voice-enabled navigation, as highlighted by Zhou et al., 2020, and AI-driven personalization, as discussed by Ramírez-Saltos et al., 2023, can dynamically adapt to diverse user needs, offering a more inclusive user experience [22,45]. Jain et al., 2025, also discussed the growing role of AI-enabled personalization, assistive technologies, and adaptive digital health platforms in supporting individuals with disabilities through more accessible and user-centered healthcare experiences [44].

Recent research from Elechi et al., 2025, has also highlighted the role of AI, particularly large language models, in clinical workflows for documentation and data processing. By reducing system complexity and clinician workload, these technologies can contribute to more streamlined and user-friendly platforms. These improvements can indirectly enhance usability in digital healthcare systems when integrated effectively, supporting accessibility [46].

Additionally, the scope of accessibility criteria must expand to accommodate next-generation platforms, including augmented reality (AR), virtual reality (VR), and Internet of Things (IoT) devices. Research by Yang et al., 2024, and Malik et al., 2024, emphasizes the importance of adaptive interfaces that can adjust to varying user preferences and device capabilities [33,35]. Similarly, culturally inclusive and privacy-conscious designs, as suggested by Maloney et al., 2020, are vital for ensuring these platforms are equitable and user-friendly [32].

Finally, as technologies advance, some traditional accessibility criteria may become less critical. Static features like fixed layouts, as noted by Gilbert, 2019, may lose relevance in dynamic and interactive interfaces [10]. Likewise, traditional input methods, such as keyboard and mouse navigation, are increasingly being replaced by touch-based and voice-driven systems, as stated by Arean et al., 2016 [28]. Future developments should prioritize adaptive, inclusive, and user-centered features to ensure accessibility remains a cornerstone of innovation.

Overall, this review contributes to the existing literature with a structured synthesis of accessibility recommendations across varying dimensions: design principles, user experience, user participation, technical considerations, among others. Previous studies have focused on specific features or disability types, whereas this review highlights how these elements collectively interact to influence accessibility in interfaces. This study offers a more comprehensive view that will support the design of healthcare systems.

## 6. Limitations

This review has several limitations. Although the review followed PRISMA guidelines, a formal review protocol was not registered. The search was limited to English-language, peer-reviewed articles from PubMed, ScienceDirect, and Web of Science. As a result, relevant studies from other sources, including IEEE Xplore and the ACM Digital Library, may not have been captured.

The included studies varied in design, sample size, and healthcare context, which may affect the generalizability of the findings. In addition, a formal quality appraisal tool was not used. Data extraction and theme identification were conducted by a single researcher, and formal inter-rater reliability measures were not calculated. Finally, publication bias may be present, as studies with significant findings are more likely to be published. Despite these limitations, the review provides a broad overview of current accessibility recommendations for digital healthcare interfaces and highlights key areas for future research.

## 7. Conclusions

This systematic literature review highlights the critical need for accessible digital healthcare interfaces for individuals with disabilities. While significant progress has been made in integrating accessibility features into healthcare platforms, challenges such as insufficient adherence to accessibility standards, limited user-centered design practices, and inadequate support for assistive technologies persist. Addressing these challenges through the recommendations provided in this study will lead to more inclusive and effective healthcare systems, ultimately improving the quality of life for individuals with disabilities. The findings contribute to ongoing discussions about the importance of digital accessibility in healthcare, emphasizing the need for continued innovation and research in this area.

## 8. Disclosure Statement

**Figure 2:**
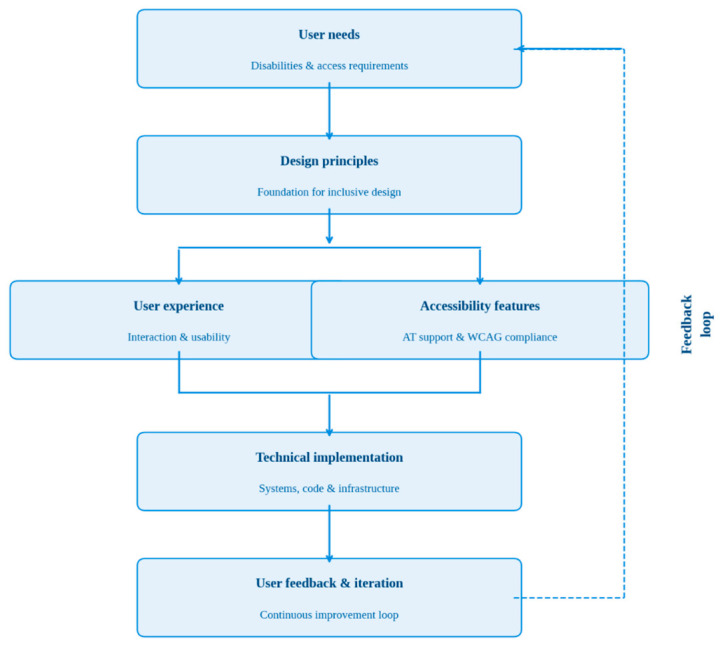
Conceptual framework illustrating key themes and relationships.

The authors report there are no competing interests to declare. No financial or non-financial interests have influenced the research presented in this systematic literature review. The authors declare that ChatGPT-5.5 was utilized for grammar correction and Claude Opus 4.8 was used for image creation in Figure 1 and Figure 2.

## Figures and Tables

**Table 1:** Search strings and keywords.

Search Strings
((“Disability” OR “Disable” OR “Disabilities” OR “Disabled” OR “Impairments” OR “Special needs”) AND (“Health” OR “Healthapps” OR “Health platforms” OR “Health portals” OR “Medical apps” OR “Patient portals”) AND (“User interface” OR “UI” OR “Interface design”)

**Table 2:** Inclusion and exclusion criteria.

Inclusion Criteria	Exclusion Criteria
Accessibility of full texts. Published in the English language. Published any time after 1 January 2014. Peer reviewed articles.	Articles that are not directly related. Full texts of the articles were not accessible.

These criteria were selected to ensure that the included studies were relevant and aligned with the objectives of this review. Limiting the selection to peer-reviewed, English-language articles helped maintain consistency, while the selected time frame focused on recent developments in digital healthcare and accessibility. Excluding unrelated studies ensured that the analysis remained focused on interface design recommendations for individuals with disabilities.

**Table 3:** Legend for Quality Assessment Ratings.

Rating	Description
High	The study strongly meets the criterion and provides clear, directly useful evidence for the review.
Moderate	The study partially meets the criterion or provides useful evidence with some limits in scope, detail, or generalizability.
Low	The study weakly meets the criterion or provides limited detail or only indirect evidence for the review.

**Table 4:** Quality Assessment of Included Studies.

Article Author	Relevance	Methodological Clarity	Contribution
Van Cleave et al., 2022 [11]	High	High	High
Garling & Stewart, 2024 [12]	High	Moderate	Moderate
Hemsley et al., 2018 [13]	High	Moderate	Moderate
Onyeaka et al., 2022 [14]	High	High	High
Ramos et al., 2021 [15]	High	Moderate	Moderate
Senjam & Primo, 2022 [16]	High	Moderate	Moderate
Buning et al., 2024 [17]	High	High	High
Jo et al., 2021 [18]	High	Moderate	Moderate
Berkowsky & Czaja, 2018 [19]	High	High	High
Van Holstein et al., 2021 [20]	High	High	High
Cosgrove et al., 2023 [21]	High	High	High
Zhou et al., 2020 [22]	High	High	High
Jabour et al., 2022 [23]	High	High	High
Fabius et al., 2023 [24]	High	High	High
Shamsujjoha et al., 2024 [25]	High	High	High
Sit et al., 2021 [26]	High	Moderate	Moderate
Bakker et al., 2018 [27]	High	High	High
Arean et al., 2016 [28]	High	High	High
Choi & Chlebek, 2024 [29]	High	Moderate	Moderate
Salgado et al., 2018 [30]	High	High	High
Steele Gray et al., 2014 [31]	High	High	High
Maloney et al., 2020 [32]	High	High	High
Yang et al., 2024 [33]	High	High	High
Campbell & Besselli, 2020 [34]	High	Moderate	Moderate
Malik et al., 2024 [35]	Moderate	Moderate	Moderate
Du & Salen Tekinbas, 2020 [36]	High	Moderate	High
Kairy et al., 2021 [37]	High	High	High
Ha et al., 2023 [38]	High	High	High
Jiam et al., 2016 [39]	High	Moderate	Moderate
Erickson, 2020 [40]	High	Moderate	Moderate
Madrigal-Cadavid et al., 2020 [41]	High	High	High
Connolly et al., 2020 [42]	High	High	High

Criteria: relevance to the research questions, methodological clarity, and overall contribution to the review objectives.

**Table 5:** Included articles in each theme.

**Accessibility Features**		
Ensure Compliance with Accessibility Standards	Following established accessibility guidelines such as WCAG (Web Content Accessibility Guidelines) ensures interfaces are usable by people with various disabilities.	[11,12,13,14,15,16,18,22,25,26,29,31,32,33,37,38,39,41,42]
User-Friendly Designs	Designing interfaces that are simple, clear, and easy to navigate, with features like large buttons, high-contrast text, and alternative text for images.	[17,18,19]
Support for Assistive Technologies	Interfaces should be compatible with screen readers, voice recognition software, and other assistive technologies to facilitate access for users with disabilities	[18,19,20,21,22]
**User Experience**		
Intuitive Navigation	Interfaces should be designed with intuitive navigation, ensuring users can easily find the information they need without unnecessary complexity.	[11,12,14,16,18,20,21,22,23,24,25,26,27,28,29,30,31,34,37,38,39,40,41,42]
Personalization Options	Providing users with the ability to customize their interface (e.g., font size, color schemes) to suit their preferences and needs can greatly enhance the user experience.	[13,14,15,17,19,21,32,33,36,41]
Feedback Mechanisms	Including mechanisms for users to provide feedback on their experience can help identify areas for continuous improvement.	[12,15,16,22,23,25,26,27,28,29,32,37,39]
**Design Principles**		
User-Centered Design	Incorporating user-centered design principles ensures that the needs and preferences of end-users, especially those with disabilities, are considered throughout the design process.	[11,12,13,14,15,16,18,19,20,21,22,24,25,26,27,28,29,30,31,32,33,34,35,36,37,38,39,40,41,42]
Consistent Layouts	Consistent use of layouts, icons, and terminology helps users understand and navigate the portal more easily.	[21]
Clear Instructions and Help Options	Providing clear, concise instructions and accessible help options can reduce user frustration and improve overall satisfaction.	[11,12,14,15,16,18,19,22,23,24,25,26,27,28,29,30,31,32,33,34,36,37,38,39,40,41]
**Technical Recommendations**		
Adaptive Interfaces	Designing adaptive interfaces that can adjust to different devices and user preferences ensures accessibility.	[11,14,15,16,19,20,21,22,23,25,26,27,28,29,30,31,32,33,35,36,37,38,39]
Robust Error Handling	Implementing robust error handling mechanisms can help users recover from mistakes.	[21,24,34,35]
Secure and Confidential	Ensuring that the portal is secure and maintains user confidentiality, especially given the sensitive nature of health information, is paramount.	[14,15,24,25,26,30,31,33,34,35,39]
**User Feedback and Participation**		
Involve Users in Design Process	Actively involving users, particularly those with disabilities, in the design and testing phases can help identify potential issues and ensure the portal meets their needs.	[13,14,15,16,17,18,20,21,22,23,24,25,26,27,28,29,30,31,32,33,34,35,36,37,39,40,41,42]
User Testing and Iteration	Conducting user testing sessions and iterating on the design based on user feedback.	[11,15,16,22,24,25,26,27,28,29,30,31,32,33,34,35,36,37,38,39,41]

## Data Availability

No new data were created or analyzed in this study.

## Appendix A. Full Database Search Strategies

### Appendix A.1. PubMed

Search string:

((“disability”[Title/Abstract] OR “disabled”[Title/Abstract] OR “disabilities”[Title/Abstract] OR “impairments”[Title/Abstract] OR “special needs”[Title/Abstract]) AND (“health”[Title/Abstract] OR “health apps”[Title/Abstract] OR “health platforms”[Title/Abstract] OR “health portals”[Title/Abstract] OR “medical apps”[Title/Abstract] OR “patient portals”[Title/Abstract]) AND (“user interface”[Title/Abstract] OR “UI”[Title/Abstract] OR “interface design”[Title/Abstract]))

Filters applied:

- English language
- Journal articles
- Publication date from 1 January 2014 to 31 December 2024

### Appendix A.2. Web of Science

Search string:

TS = ((“disability” OR “disabled” OR “disabilities” OR “impairments” OR “special needs”) AND (“health” OR “health apps” OR “health platforms” OR “health portals” OR “medical apps” OR “patient portals”) AND (“user interface” OR “UI” OR “interface design”))

Filters applied:

- English language
- Article document type
- Publication years 2014–2024

### Appendix A.3. ScienceDirect

Search string:

TITLE-ABSTR-KEY((“disability” OR “disabled” OR “disabilities” OR “impairments” OR “special needs”) AND (“health” OR “health apps” OR “health platforms” OR “health portals” OR “medical apps” OR “patient portals”) AND (“user interface” OR “UI” OR “interface design”))

Filters applied:

- Research articles
- English language
- Publication years 2014–2024
